# Midterm Clinical Impacts of Biodegradable Polymer Everolimus-Eluting Stents Compared with Durable Polymer Everolimus-Eluting Stents: A 3-Year Propensity-Matched Study

**DOI:** 10.1155/2020/2869303

**Published:** 2020-04-20

**Authors:** Hiroaki Matsuda, Ai Kagase, Takahiro Tokuda, Yusuke Ochiumi, Akira Murata, Yoriyasu Suzuki, Tatsuya Ito

**Affiliations:** Department of Cardiology, Nagoya Heart Center, Nagoya, Japan

## Abstract

**Objectives:**

Our aim was to evaluate the safety and efficacy of biodegradable polymer everolimus-eluting stents (BP-EES) compared with durable polymer everolimus-eluting stents (DP-EES) in midterm.

**Background:**

There are few data about midterm clinical outcomes of BP-EES compared with DP-EES.

**Methods and Results:**

Between January 2016 and December 2017, 395 consecutive patients were treated with BP-EES and 391 consecutive patients were treated with DP-EES in Nagoya Heart Center. The primary endpoint was a 3-year cumulative incidence of target lesion failure (TLF) defined as cardiac death, target vessel myocardial infarction (MI), and clinical indicated target lesion revascularization (TLR). Moreover, clinical indicated target vessel revascularization (TVR) and definite stent thrombosis (ST) were also evaluated as the secondary endpoints. After propensity score matching, 327 patients were selected in each group. At 3 years, the cumulative incidence of TLF was 4.5% in the BP-EES group versus 6.5% in DP-EES (adjusted HR 0.67 (95% CI 0.33–1.30), log-rank *P*=0.23). Regarding the individual components of the TLF at 3 years, the cumulative incidence of target vessel MI was significantly lower in BP-EES than in DP-EES (0% versus 1.9%: adjusted HR 0.83 (95% CI 0.71–0.97), log-rank *P*=0.01), but there was no difference between BP-EES and DP-EES in the incidence of cardiac death and clinically indicated TLR. The cumulative 3-year incidence of definite ST was significantly lower in BP-EES than in DP-EES (0% versus 1.6%, log-rank *P*=0.02).

**Conclusions:**

There were no significant differences of TLF between BP-EES and DP-EES within 3 years. In this study, BP-EES seems to prevent definite ST and be safer than DP-EES in midterm.

## 1. Introduction

The everolimus-eluting stents (EES) that deliver antiproliferative everolimus analogue from durable polymer (DP) were associated with superior safety and efficacy outcomes compared with the first-generation drug-eluting stents (DES) [1, 2]. However, DP provokes some inflammation, delayed neointimal healing, and incomplete endothelialization, [3] which might cause the risk of late catch-up events such as late stent thrombosis (ST) and restenosis.

The biodegradable polymer EES (BP-EES) were developed with the hope of providing similar safety clinical outcomes to bare metal stents (reduced risk of ST), while maintaining an efficacy profile of DP-EES (reduced risk of target lesion revascularization). Currently, several papers have described the long-term outcome using BP-EES, [4, 5] but there are few data clinical outcomes of BP-EES compared with DP-EES.

The objective of the current study was to evaluate the safety and efficacy of BP-EES compared with DP-EES in the midterm.

## 2. Methods

### 2.1. Study Population

**Figure 1:**
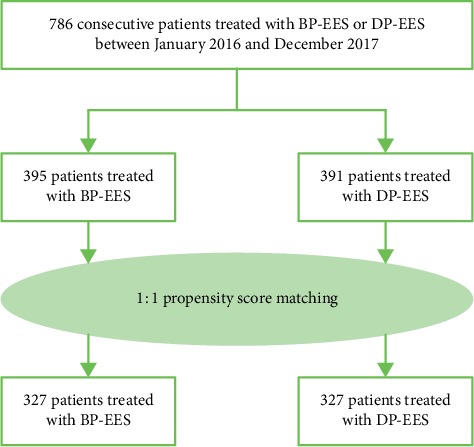
Study flow chart.

This is a retrospective, observational, and single-center study enrolling patients after successful BP-EES or DP-EES implantation. Between January 2016 and December 2017, 786 consecutive patients underwent percutaneous coronary intervention (PCI) with BP-EES (395 patients) or DP-EES (391 patients) at Nagoya Heart Center. After propensity score matching, 327 patients were selected in each group (Figure 1). All patients gave written informed consent for the procedure and the follow-up protocol, which was approved by the ethics committee of our hospital.

### 2.2. Procedures

The PCI strategy was left to the discretion of the operating surgeon. Patients who were scheduled for PCI received oral daily administration of aspirin (≥81 mg/day) and P2Y12 inhibitor (75 mg/day clopidogrel or 3.75 mg/day plasugrel). Ticlopidine 200 mg/day was only allowed for those who did not tolerate clopidogrel and plasugrel. Patients with acute coronary syndromes (ST-elevation myocardial infarction and NSTE-acute coronary syndrome) received loading doses of aspirin (200 mg) and P2Y12 inhibitor (300 mg clopidogrel or 20 mg/day plasugrel). During the procedure, unfractionated heparin (100 U/kg) was administrated to all the patients in order to achieve an activated clotting time of 250 seconds were used according to the operator's judgement. After the procedure, all the patients were recommended to receive optimal pharmacologic therapy including statins, beta-blockers, or renin-angiotensin system blockade following the current guidelines. Moreover, duration of dual antiplatelet therapy (DAPT) also depended on the operator's discretion.

### 2.3. Data Collection and Follow-Up

All the patients were followed up at 1, 3, 6, and 12 months after their index procedure and annually thereafter. Additional information was obtained by telephone contact or medical records, if necessary. A follow-up angiography 6 to 12 months after stent implantation was recommended to the patients according to clinical symptoms and findings.

### 2.4. Endpoints and Definitions

The primary endpoint in this study was target lesion failure (TLF) at 3 years after the index procedure, defined as a composite of cardiac death, target vessel myocardial infarction (MI), and clinically indicated target lesion revascularization (TLR). The secondary endpoint included the individual components of the composite primary endpoints and definite stent thrombosis (ST) at various time points.

Death was considered as cardiac unless an unequivocal noncardiac cause could be established. MI and ST were defined according to the Academic Research Consortium definitions [6]. TLR was defined as either PCI or coronary artery bypass grafting due to restenosis or thrombosis of the target lesion that included the proximal and distal edge segments (within 5 mm) as well as the ostium of the side branches. Clinically driven TLR was defined as TLR performed because of ischaemic symptoms, electrocardiographic changes at rest, or positive stress test results.

## 3. Statistical Analysis

Categorical variables were presented as number and percentage and continuous variables were expressed as mean value ± SD or median with interquartile range. Cumulative incidence was estimated by the Kaplan–Meier method and differences were assessed with the log-rank test. To evaluate the late events beyond 1 year, we used landmark analysis at 1 year. Those patients with the individual endpoint events before 1 year were excluded in the landmark analysis. We then included them simultaneously in the multivariable models and obtained the adjusted hazard ratios and their 95% confidence intervals. To match the patients for various clinical and angiographic characteristics, we used the propensity score matching method using a multivariate logistic regression model. The selected variables included demographics (gender and age), clinical presentation, comorbidities at baseline, prior treatment (PCI or CABG), angiographic and procedural characteristics, and medications administered at discharge. The patients were matched in a 1 : 1 ratio on the propensity score; we did an exact match for region and used a 5% caliper matching for the propensity score for the other variables [7]. The matching was deemed satisfactory when the standardized mean differences were less than 10%. Statistical analysis was performed with the use of JMP version 14.0 (SAS Institute Inc., Cary, NC, USA). A 2-sided *P* value of <0.05 was considered statistically significant.

## 4. Results

### 4.1. Baseline Characteristics

Baseline characteristics were not significantly different between the BP-EES and DP-EES groups after propensity score matching (Table 1). The median follow-up duration of the BP-EES group was 1068 (first and third quartiles (Q1–Q3): 885–1285) days, while that of the DP-EES group was 1037 (first and third quartiles (Q1–Q3): 841–1239) days. Moreover, the 3-year clinical follow-up of the BP-EES group was completed in 318 patients (97.2%) among 327 patients, while that of the DP-EES group was completed in 323 patients (98.7%) among 327 patients.

### 4.2. Clinical Outcomes

**Figure 2:**
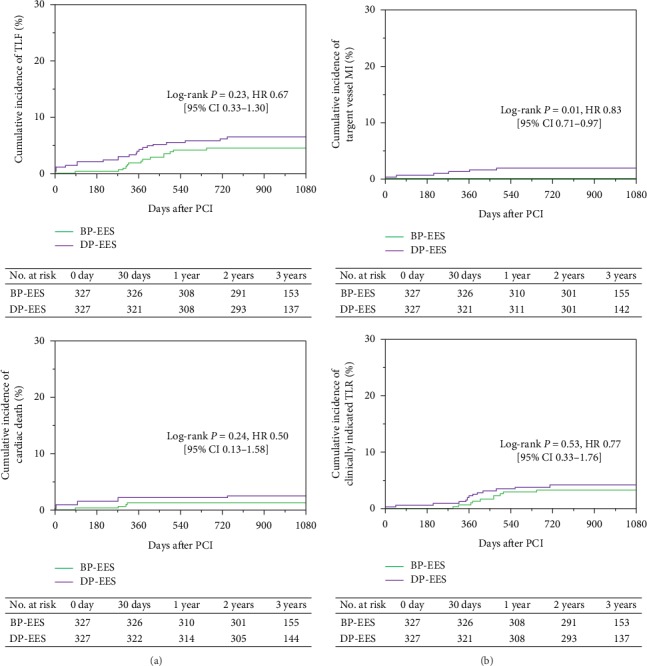
Cumulative incidence of the primary endpoint and its individual components at 3 years.

**Figure 3:**
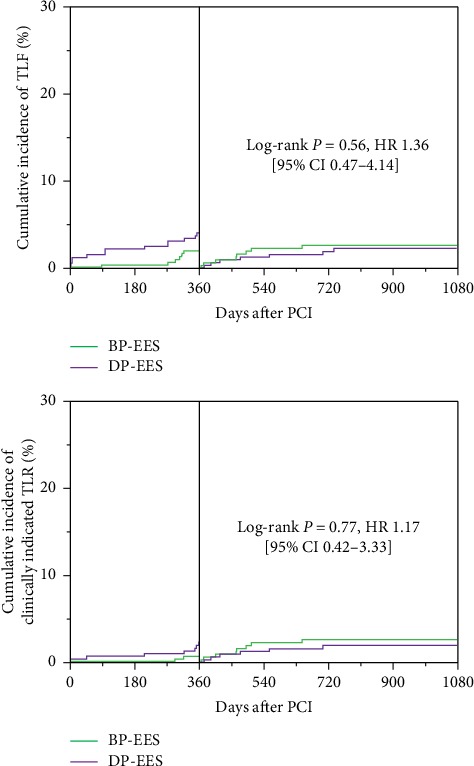
Cumulative incidence of the TLF and clinically indicated TLR at 1-year landmark analysis.

At 3 years, the device-oriented composite clinical endpoint TLF occurred in 14 of 327 patients (4.5%) assigned to BP-EES and 21 of 327 patients (6.5%) assigned to DP-EES (BP-EES versus DP-EES: adjusted HR 0.67 (95% CI 0.33–1.30), log-rank *P*=0.23). However, the cumulative 1-year incidence of the TLF tended to be lower in BP-EES than in DP-EES (1.9% versus 4.3%, log-rank *P*=0.07) (Figure 2 and Table 2). Regarding the individual components of the TLF at 3 years, the cumulative incidence of target vessel MI was significantly lower in BP-EES than in DP-EES (0% versus 1.9%: adjusted HR 0.83 (95% CI 0.71–0.97), log-rank *P*=0.01), but there was no difference between BP-EES and DP-EES in the incidence of cardiac death and clinically indicated TLR (Figure 2 and Table 2). Landmark analyses between 1-year and 3-year follow-up (Figure 3 and Table 2) showed no difference in TLF and its individual components between BP-EES and DP-EES.

**Figure 4:**
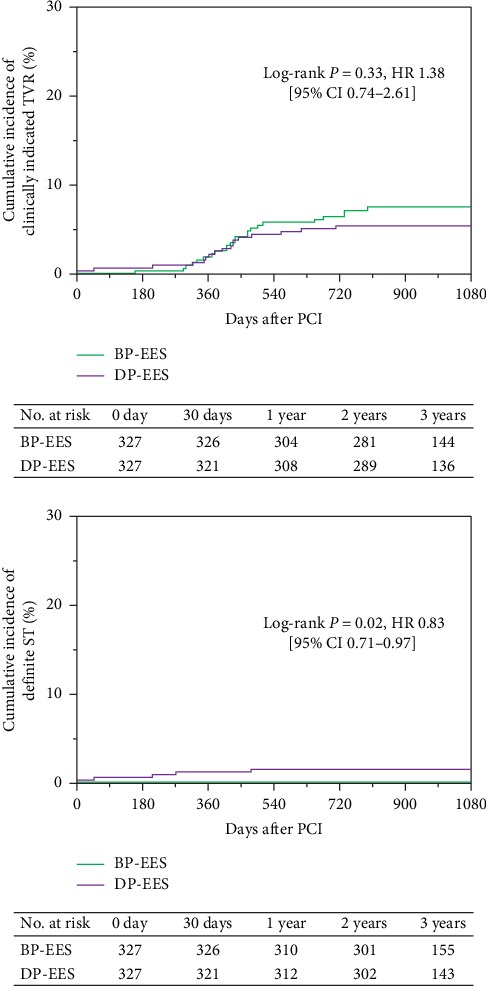
Cumulative incidence of the secondary endpoints.

Regarding the secondary endpoints, the cumulative 3-year incidence of definite ST was significantly lower in BP-EES than in DP-EES (0% versus 1.6%, log-rank *P*=0.02), while that of clinically indicated TVR was not significantly different between the 2 groups (Figure 4 and Table 2). The cumulative 1-year incidence of definite ST was also significantly lower in BP-EES than in DP-EES (0% versus 1.3%, log-rank *P*=0.04) (Figure 4 and Table 2).

## 5. Discussion

The main findings of the current study are that compared with DP-EES, use of BP-EES was associated (1) with similar efficacy regarding TLF; (2) with superior safety regarding definite ST reduction in midterm.

Several randomized control trials and recent meta-analysis study comparing BP-DES with DP-DES showed that BP-DES have similar efficacy profiles to DP-DES [4, 8].

In this study, BP-EES proved to be comparable to DP-EES for TLF at 3 years. The rates of cardiac death, clinically indicated TLR and TVR up to 3 years were similar for the both stents. The thickness of the stent strut strongly was reported to influence the incidence of stent restenosis [9]. Compared with thinner struts, thicker strut platforms have been shown to increase platelet aggregation and inflammatory cell adhesion, [10] which might provoke in-stent hyperplasia caused by stent restenosis. The BP-EES strut thickness (2.25–2.75 mm;74 *μ*m, 3.0–3.5 mm;79 *μ*m, 4.0 mm; 81 *μ*m) is as thin as DP-EES (2.25–4.0 mm;81 *μ*m), which might demonstrate the similar efficacy outcomes for the both stents regarding TLR in particular.

**Figure 5:**
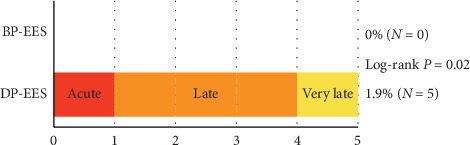
Definite ST through 3 years.

However, the rates of target vessel MI and definite ST up to 3 years in BP-EES was significantly lower than DP-EES. The main cause of target vessel-related MI in this study was mostly involved in definite ST. The widespread use of the DP-DES has not resolve the late catch-up events such as late ST and very late ST. In the present day, late ST and very late ST are rare events, but sometimes threat life. Recently, several large-scale first-generation DES registries have demonstrated that the annual incidences of ST were 0.21 to 0.53% per year [11–15]. The DES is reported to be less thrombogenic compared with BMS by the bench testings [10]. Regarding second-generation DES, the rates of late and very late ST were consistently very low after implantation of the DP-EES in particular in clinical trials [16, 17]. Though the cumulative incidence of definite ST for the DP-EES was not so high, it was noteworthy that definite ST never occurred in patients after BP-EES implantation in this study. More importantly, there was a significant difference in late ST between BP-EES and DP-EES (0% versus 1.6%, log-rank *P*=0.02) (Figure 5 and Table 2). The ST was reported to depend on malapposition of the deployed stent or internal use of antiplatelet agent [18]. The DAPT was continued up to 6 months by all the eligible patients and changed for that of single antiplatelet agent 6 months after performing PCI as mentioned earlier. Only one late ST case received DAPT, but the other ST cases took single antiplatelet agent according to the ESC-guideline. Salvatore De Rosa et al. suggests that prolongation of DAPT, as well as the use of newer P2Y12 antagonists could have contributed to the better performance of PCI and could help the further improvement of the clinical outcome after PCI of the left main coronary artery [19]. The late or very late ST might be link DAPT discontinuation. Also, all the ST cases in this study were assessed by the qualitative coronary angiography (QCA) or intravascular ultrasound (IVUS). An acute ST case was performed with long stenting for sublumen. Subacute ST case was none in this study. Three late ST and one very late ST cases have optimal stenting from the findings of the QCA and IVUS. In the BP-EES, the polymer is absorbed and which is resorbed within 4 months, this is because the BP-EES is designed to enhance stent healing with everolimus-eluting polymer applied only to the abluminal stent surface [20, 21]. The results of our study may suggest that the biocompatibility of BP-EES may be involved in preventing of late ST and very late ST.

There are several important limitations in this study. First, this study was a retrospective, single-centre study, which may have significantly affected some results due to confounding factors. Although we performed propensity score matching analysis to adjust the potential confounding factors, we did not correct for all possible and unmeasured variables. Therefore, the results of the current study should be considered as hypothesis generating only, being a post-hoc analysis of a trial. Second, the clinical event rates of BP-EES or DP-EES were relatively low and the power of the present study was inadequate to draw any definite conclusion, especially for stent thrombosis. This limitation might be originated by use of imaging device for the most patients. Third, we did not have information on bleeding complications during the follow-up. Finally, current follow-up duration is limited to only 3 year. Indeed, longer follow-up in more complex patient/lesion subsets may better differentiate between stent platforms with different structural design or polymer-healing attributes.

## 6. Conclusions

In conclusion, the 3-year cumulative incidence of TLF had no significant differences between BP-EES and DP-EES. In this study, BP-EES might prevent definite ST and be safer than DP-EES in the midterm.

## Figures and Tables

**Table 1:** Baseline characteristics before and after propensity score matching.

	Before PSM			After PSM		
	BP-EES	DP-EES	*P* value	BP-EES	DP-EES	*P* value
Age (years)	68.7 ± 10.2	69.4 ± 10.3	0.31	68.9 ± 10.0	69.4 ± 10.4	0.37
Age *t* ≥ 75 years	116 (29%)	124 (32%)	0.28	98 (30%)	110 (34%)	0.31
Male gender	284 (72%)	297 (76%)	0.17	238 (73%)	249 (76%)	0.32
Body mass index	23.9 ± 3.3	24.1 ± 3.5	0.57	23.9 ± 3.4	24.1 ± 3.5	0.56
Hypertension	284 (72%)	278 (71%)	0.74	236 (72%)	232 (71%)	0.73
Dyslipidemia	265 (67%)	242 (62%)	0.31	217 (66%)	205 (63%)	0.33
Diabetes mellitus	150 (38%)	168 (43%)	0.30	123 (38%)	136 (42%)	0.30
Insulin-treated diabetes	19 (5%)	25 (6%)	0.33	14 (4%)	17 (5%)	0.58
Treated with oral medication only	104 (21%)	90 (23%)	0.61	72 (22%)	75 (23%)	0.78
Treated with diet therapy only	47 (12%)	39 (10%)	0.70	35 (11%)	33 (10%)	0.80
Current smoker	91 (23%)	78 (20%)	0.31	72 (22%)	65 (20%)	0.31
Creatinine (mg/dL)	1.32 ± 1.71	1.74 ± 2.26	0.08	1.37 ± 1.75	1.51 ± 2.07	0.33
Hemodialysis	26 (7%)	43 (11%)	0.03	24 (7%)	29 (9%)	0.47
Ejection fraction (%)	58.0 ± 11.3	56.8 ± 12.1	0.24	57.7 ± 11.1	57.3 ± 11.9	0.63
Clinical presentation						
Stable coronary artery disease	345 (87%)	360 (92%)	0.03	288 (88%)	296 (91%)	0.31
Unstable angina	15 (4%)	8 (2%)	0.15	13 (4%)	8 (2%)	0.27
Acute myocardial infarction	36 (9%)	23 (6%)	0.09	26 (8%)	23 (7%)	0.66
Prior myocardial infarction	63 (16%)	90 (23%)	0.03	63 (19%)	66 (20%)	0.77
Prior percutaneous coronary intervention	119 (30%)	145 (37%)	0.04	104 (32%)	118 (36%)	0.25
Prior coronary artery bypass grafting	7 (2%)	16 (5%)	0.02	7 (2%)	12 (4%)	0.25
Prior stroke	28 (7%)	31 (8%)	0.40	22 (7%)	27 (8%)	0.46
Peripheral vascular disease	43 (11%)	57 (15%)	0.15	37 (11%)	37 (11%)	1.0
Atrial fibrillation	28 (7%)	35 (9%)	0.17	23 (7%)	26 (8%)	0.66
Medications						
Aspirin	395 (100%)	390 (99.7%)	0.32	327 (100%)	326 (99.7%)	0.32
Thienopyridines	394 (99.7%)	390 (99.7%)	0.31	326 (99.7%)	327 (100%)	0.32
Prasugrel	104 (26.3%)	84 (25.7%)	0.21	89 (27.2%)	84 (25.7%)	0.66
Clopidogrel	235 (72.7%)	235 (74.9%)	0.47	234 (71.6%)	235 (71.9%)	0.93
Ticlopidine	3 (0.8%)	10 (2.6%)	0.05	3 (0.9%)	9 (2.8%)	0.08
Anticoagulants	21 (5.3%)	31 (7.9%)	0.14	19 (5.8%)	24 (7.3%)	0.43
Warfarin	10 (2.5%)	17 (4.3%)	0.16	9 (2.8%)	12 (3.7%)	0.51
Direct-acting oral anticoagulants	12 (2.8%)	12 (3.6%)	0.53	10 (3.1%)	12 (3.7%)	0.67
B-blockers	165 (42%)	163 (42%)	0.98	141 (43%)	137 (42%)	0.75
ACE-I/ARB	216 (55%)	223 (57%)	0.51	184 (56%)	195 (60%)	0.22
Calcium-channel blockers	173 (44%)	163 (42%)	0.55	146 (45%)	131 (40%)	0.24
Statins	302 (76%)	271 (69%)	0.02	250 (76%)	233 (71%)	0.13
Lesion and procudural characteristics						
Target vessel location						
Left main coronary artery	7 (2%)	11 (3%)	0.15	6 (2%)	8 (3%)	0.31
Left anterior descending coronary artery	183 (46%)	206 (53%)	0.06	160 (49%)	176 (54%)	0.21
Left circumflex coronary artery	85 (22%)	84 (21%)	0.99	72 (22%)	73 (22%)	0.93
Right coronary artery	120 (30%)	90 (23%)	0.02	89 (27%)	70 (21%)	0.08
Bypass graft	0 (0%)	0 (0%)	1.0	0 (0%)	0 (0%)	1.0
Number of treated lesions per patient	1.82 ± 0.74	1.83 ± 0.74	0.80	1.84 ± 0.75	1.81 ± 0.73	0.60
Ostium	35 (9%)	43 (11%)	0.51	30 (9%)	35 (11%)	0.51
Bifurcation	147 (37%)	130 (33%)	0.24	128 (39%)	108 (33%)	0.12
Diffuse lesion (lesion length >20 mm)	174 (44%)	160 (41%)	0.50	141 (43%)	135 (41%)	0.45
Severe calcification	55 (14%)	39 (10%)	0.08	42 (13%)	34 (10%)	0.33
Chronic total occlusion	33 (8%)	38 (10%)	0.50	28 (9%)	30 (9%)	0.78
In-stent restenosis	17 (4%)	25 (6%)	0.19	16 (5%)	17 (5%)	0.86
Number of stents used per patient	1.17 ± 0.43	1.11 ± 0.36	0.04	1.17 ± 0.43	1.11 ± 0.37	0.10
Stent diameter (mm)	2.97 ± 0.50	3.06 ± 0.47	<0.01	2.97 ± 0.50	3.04 ± 0.47	0.08
Total stent length per patient (mm)	28.0 ± 14.6	26.4 ± 13.7	0.10	28.3 ± 14.6	26.2 ± 14.1	0.06
Imaging device used	394 (99.7%)	391 (100%)	0.32	326 (99.7%)	327 (100%)	0.32
Rotablator used	47 (12%)	38 (10%)	0.33	32 (10%)	32 (10%)	1.0

Values are expressed as mean ± SD or number (%). ACE-I: angiotensin converting enzyme inhibitors. ARB: angiotensin II receptor blockers.

**Table 2:** Clinical outcomes at 1 year and 3 years.

	No. of patients with at least one event (cumulative incidence)		*P* value
	BP-EES *N* = 327	DP-EES *N* = 327	
Until 1-year follow-up			
Target lesion failure	6 (1.9%)	14 (4.3%)	0.07
Cardiac death	4 (1.3%)	7 (2.2%)	0.36
Target vessel myocardial infarction	0 (0%)	5 (1.6%)	0.02
Clinically indicated target lesion revascularization	2 (0.6%)	7 (2.2%)	0.10
Clinically indicated target vessel revascularization	6 (1.9%)	7 (2.2%)	0.79
Definite stent thrombosis	0 (0%)	4 (1.3%)	0.04

Until 3-years follow-up			
Target lesion failure	14 (4.5%)	21 (6.5%)	0.23
Cardiac death	4 (1.3%)	8 (2.5%)	0.24
Target vessel myocardial infarction	0 (0%)	6 (1.9%)	0.01
Clinically indicated target lesion revascularization	10 (3.2%)	13 (4.1%)	0.53
Clinically indicated target vessel revascularization	23 (7.5%)	17 (5.5%)	0.33
Definite stent thrombosis	0 (0%)	5 (1.6%)	0.02

Landmark analysis between 1–3 year			
Target lesion failure	8 (2.6%)	7 (2.3%)	0.77
Cardiac death	0 (0%)	1 (0.3%)	0.31
Target vessel myocardial infarction	0 (0%)	1 (0.3%)	0.32
Clinically indicated target lesion revascularization	8 (2.6%)	6 (1.9%)	0.56
Clinically indicated target vessel revascularization	17 (5.6%)	10 (3.2%)	0.16
Definite stent thrombosis	0 (0%)	1 (0.3%)	0.32

## Data Availability

No data were used to support this study.
